# Recurrent Processing during Object Recognition

**DOI:** 10.3389/fpsyg.2013.00124

**Published:** 2013-04-01

**Authors:** Randall C. O’Reilly, Dean Wyatte, Seth Herd, Brian Mingus, David J. Jilk

**Affiliations:** ^1^Department of Psychology and Neuroscience, University of Colorado BoulderBoulder, CO, USA; ^2^eCortex, Inc.Boulder, CO, USA

**Keywords:** object recognition, computational model, recurrent processing, feedback, winners-take-all mechanism

## Abstract

How does the brain learn to recognize objects visually, and perform this difficult feat robustly in the face of many sources of ambiguity and variability? We present a computational model based on the biology of the relevant visual pathways that learns to reliably recognize 100 different object categories in the face of naturally occurring variability in location, rotation, size, and lighting. The model exhibits robustness to highly ambiguous, partially occluded inputs. Both the unified, biologically plausible learning mechanism and the robustness to occlusion derive from the role that recurrent connectivity and recurrent processing mechanisms play in the model. Furthermore, this interaction of recurrent connectivity and learning predicts that high-level visual representations should be shaped by error signals from nearby, associated brain areas over the course of visual learning. Consistent with this prediction, we show how semantic knowledge about object categories changes the nature of their learned visual representations, as well as how this representational shift supports the mapping between perceptual and conceptual knowledge. Altogether, these findings support the potential importance of ongoing recurrent processing throughout the brain’s visual system and suggest ways in which object recognition can be understood in terms of interactions within and between processes over time.

## Introduction

One of the most salient features of the mammalian neocortex is the structure of its connectivity, which provides for many forms of *recurrent* processing, where neurons mutually influence each other through direct, bidirectional interactions. There are extensive bidirectional excitatory and inhibitory connections within individual cortical areas, and almost invariably, every area that receives afferent synapses from another area, also sends back efferent synapses in return (Felleman and Van Essen, 1991; Scannell et al., 1995; Sporns and Zwi, 2004; Sporns et al., 2007). We describe an explicit computational model (*LVis* – Leabra Vision) of the function of this recurrent architecture in the context of visual object recognition, demonstrating a synergy between the learning and processing benefits of recurrent connectivity.

Recurrent processing, for example, has been suggested to be critical for solving certain visual tasks such as figure-ground segmentation (Hupe et al., 1998; Roelfsema et al., 2002; Lamme and Roelfsema, 2000), which requires integration of information from outside the classical receptive field. We demonstrate how recurrent excitatory processing could provide a similar function in visual occlusion, which requires the organization of image fragments that span multiple receptive fields into a logical whole *Gestalt* and involves the filling-in of missing visual information (Kourtzi and Kanwisher, 2001; Lerner et al., 2002; Rauschenberger et al., 2006; Weigelt et al., 2007; Wyatte et al., 2012a).

At a more local level, recurrent inhibitory processing produces sparse distributed representations, implemented in LVis through the use of a k-Winners-Take-All (kWTA) inhibition function (where *k* represents the roughly 15–25% activity levels present in neocortical networks; O’Reilly, 1998; O’Reilly and Munakata, 2000; O’Reilly et al., 2012). The sparse distributed representations produced by these recurrent inhibitory dynamics have been shown to produce biologically realistic representations in response to natural stimuli (e.g., O’Reilly and Munakata, 2000; Olshausen and Field, 2004; O’Reilly et al., 2012). We show here that inhibitory recurrent dynamics and sparse distributed representations make our model more robust in the face of ambiguity, by testing recognition performance with occluded visual inputs.

In the non-human primate neuroanatomy, object recognition involves the flow of visual information through the ventral pathway, originating in primary visual cortex (V1), continuing through extrastriate areas (V2, V4), and terminating in inferotemporal (IT) cortex (Hubel and Wiesel, 1962; Van Essen et al., 1992; Ungerleider and Haxby, 1994). IT neurons exhibit robust object-level encoding over wide ranges of position, rotation, scale, and lighting variability (Logothetis et al., 1995; Tanaka, 1996; Riesenhuber and Poggio, 2002; Rolls and Stringer, 2006; Tompa and Sary, 2010; DiCarlo et al., 2012). Object recognition in the human cortex operates in a similar hierarchical fashion, with homologous object-selective regions distributed throughout the lateral occipital cortex (LOC) (Grill-Spector et al., 2001; Orban et al., 2004; Kriegeskorte et al., 2008).

Computational models of object recognition that implement a feedforward, hierarchical version of the ventral pathway have explained many aspects of the initial neural response properties across these different brain areas (Fukushima, 1980, 2003; Wallis and Rolls, 1997; Riesenhuber and Poggio, 1999; Masquelier and Thorpe, 2007). Furthermore, when coupled with a supervised learning procedure (e.g., support vector machines), these models perform well at challenging computational tests of object recognition (Fei-Fei et al., 2007; Serre et al., 2007c; Mutch and Lowe, 2008; Pinto et al., 2009). Thus, they establish that primarily feedforward-driven neural responses properties based on the initial responses of the ventral pathway are *sufficient* to solve reasonably challenging versions of the object recognition problem (Serre et al., 2007a,b; DiCarlo et al., 2012).

The LVis model builds upon this feedforward processing foundation, and learns a very similar hierarchical solution to the object recognition problem. In our tests on 100-way object classification with reasonable levels of variability in location, rotation, size, and lighting, LVis performs in the same general range as these established feedforward models. Interestingly, it does so using a single unified, biologically based learning mechanism that leverages bidirectional recurrent processing between layers, to enable signals from other modalities and brain areas to shape visual object recognition during learning in important ways, supporting a form of error-driven learning (O’Reilly, 1996; O’Reilly and Munakata, 2000; O’Reilly et al., 2012). Error-driven learning is almost certainly essential for solving hard computational problems (O’Reilly and Munakata, 2000; Hinton and Salakhutdinov, 2006), and is a central element in all of the above high performance object recognition systems at the supervised learning stage. Furthermore, there are indications that error-driven learning is actually doing most of the work in object recognition models, as good performance is possible even with random visual filters (Jarrett et al., 2009).

The recurrent connectivity in our LVis model leads to a clear prediction: representations in other brain areas that project into the object recognition pathway should shape the way it develops through learning. Recent evidence indeed suggests that neurons in IT cortex reflect significant higher-level “semantic” influences, in addition to the expected stimulus-driven similarities among objects (Kiani et al., 2007; Kriegeskorte et al., 2008; Mahon and Caramazza, 2011). We show that recurrent processing within our model provides a satisfying account of this data. Furthermore, we show how recurrent processing provides a mechanism via which this higher-level semantic information can be integrated with visual information during object processing (Lupyan and Spivey, 2008; Lupyan et al., 2010; Lupyan, 2012), providing a mapping between perceptual and conceptual representations (Gotts et al., 2011).

**Figure 1:**
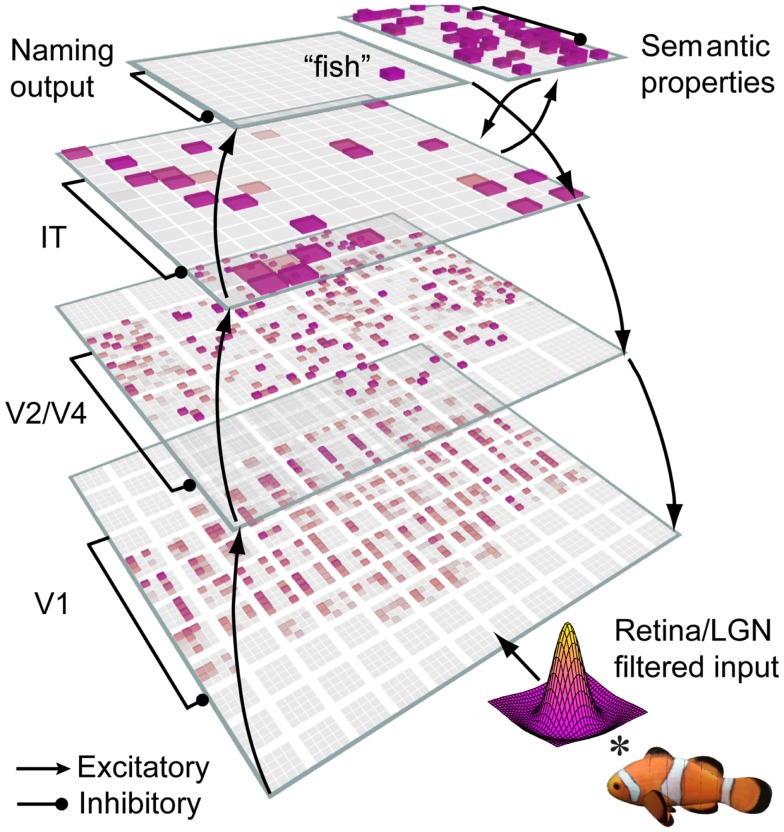
**Architecture of the LVis model**. The LVis model is based on the anatomy of the ventral pathway of the brain, from primary visual cortex (V1) through extrastriate areas (V2, V4) to inferotemporal (IT) cortex. V1 reflects filters that model the response properties of V1 neurons (both simple and complex subtypes). In higher-levels, receptive fields become more spatially invariant and complex, reflecting organizational influence from non-visual properties like semantics. All layers are reciprocally connected, allowing higher-level information to influence bottom-up processing during both the initial learning and subsequent recognition of objects, and contain local, recurrent inhibitory dynamics that limit activity levels across layers.

Altogether, we argue that this model provides an integration of diverse sources of data on the object recognition system and shows how a small, unified set of biological mechanisms can potentially solve one of the most difficult and important computational problems that the brain is known to solve (Marr, 1982; Pinto et al., 2008). Our recurrent model (Figure 1) embodies these ideas, and provides one way of extending our understanding of object recognition beyond the initial, feedforward-driven responses.

Despite the multiple influences of recurrent processing cited above, it also might not confer performance advantages in all object recognition tasks. For example, objects presented isolated and intact, without any source of degradation or ambiguity could reasonably be resolved via feedforward processing. And indeed, recurrent processing during relatively simple tasks has actually been shown to incur small costs in raw performance, because small errors in processing can become magnified over the course of repeated recurrent interactions (O’Reilly, 2001). These small costs, however, can pay dividends in more difficult object recognition problems involving occlusion or generalization across non-visual, semantic dimensions such as during semantic inference.

In short, our model provides a possible synthesis in the debate about the relative contributions of feedforward and recurrent processing in vision (Lamme and Roelfsema, 2000; Kveraga et al., 2007; Vanrullen, 2007; Roland, 2010). For well-learned, unambiguous stimuli, object recognition can operate rapidly in a feedforward-dominant manner, consistent with rapid visual processing in some experiments (Thorpe et al., 1996; VanRullen and Koch, 2003; Liu et al., 2009). This feedforward-dominant processing can be observed directly in the dynamics of our model as we show below. However, the extensive recurrent connectivity found throughout the ventral pathway can also play an important function in forming robust representations needed for more complex object recognition problems that involve ambiguity, such as when objects are occluded. This translates to longer overall latencies for the recognition decision, but with the added benefit of a coherent and robust interpretation of a visual scene that arises from the integration of signals at different levels of the hierarchy (Lamme and Roelfsema, 2000; Kveraga et al., 2007; Roland, 2010).

## Results

### Object recognition dataset

**Figure 2:**
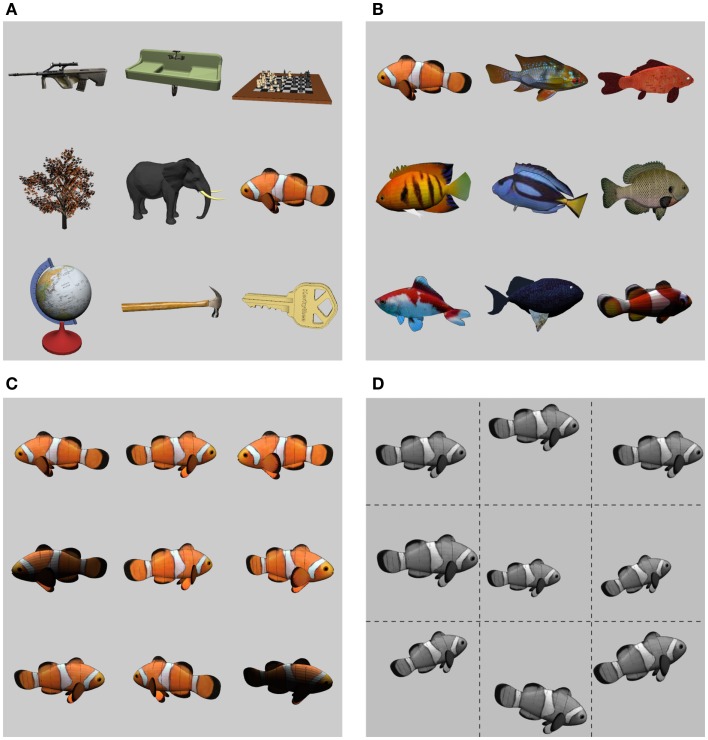
**The *CU3D-100* dataset**. **(A)** Nine example objects from the 100 CU3D categories. **(B)** Each category is further composed of multiple, diverse exemplars (average of 9.42 exemplars per category). **(C)** Each exemplar is rendered with 3D (depth) rotations and variability in lighting. **(D)** In training and testing the models described here, the 2D images were converted to grayscale and subjected to 2D transformations (translation, scale, planar rotation), with ranges generally around 20%.

Before exploring the ways in which recurrent processing impacts the dynamics of object recognition, we briefly describe the basic set of objects on which the network was trained and tested, which we call the *CU3D-100* dataset^1^. CU3D-100 is organized into 100 categories with an average of 9–10 exemplars per category and controlled variability in pose and illumination (Figures 2A–D). The dataset was designed to address problems with existing datasets based on naturalistic images, such as the Caltech101 (Ponce et al., 2006; Pinto et al., 2008). Naturalistic image datasets, while useful for benchmarking the ability of object recognition systems on realistic visual stimuli, are often underconstrained for studying biological principles of object recognition such as invariance or the recurrent processing effects that are of interest here. This is because object exemplars are often present in a fixed pose and with additional background clutter that is can be correlated with the object’s category, and foreground and background image elements cannot be independently manipulated. The CU3D-100 dataset, in contrast, uses a “synthetic” approach in which object models and backgrounds *can* be controlled independently and then rendered to bitmap images, allowing an experimenter to isolate and gain full control over the parameters that govern the core challenge of the object recognition problem (Pinto et al., 2008, 2009, 2011; DiCarlo et al., 2012). Datasets that use 3D models are gaining popularity in the literature, but are labor-intensive to create, and thus usually only consist of a handful of object categories and exemplars (e.g., LeCun et al., 2004). To our knowledge, this is the first synthetic dataset that approaches the size and scope of larger benchmark datasets like Caltech101.

For the purposes of the present research, we rendered the object models against uniform backgrounds as opposed to cluttered backgrounds. Although background clutter is clearly more relevant for real-world applications of object recognition, we think that it is not realistic from a biological perspective to assume that the upper levels of the ventral visual pathway (V4 and IT) have to contend with the full impact of this background clutter. This is because extensive research has indicated that early levels of the visual pathway, specifically in area V2, contain specialized figure-ground processing mechanisms that perform border ownership labeling (Zhaoping, 2005; Craft et al., 2007; Poort et al., 2012). Thus, features belonging to the background are not grouped with those associated with the foreground object, and this filtering process enables higher-level areas to perform spatial and featural integration processes without suffering as much interference from irrelevant background features as would otherwise be the case in a model lacking these figure-ground filtering mechanisms. Consistent with this perspective, various sources of data indicate that IT represents relevant objects without significant interference from irrelevant background clutter (Baylis and Driver, 2001; Kourtzi and Kanwisher, 2001; Lerner et al., 2002).

Thus, our goal with the present simulations was to enable the model to achieve high levels of performance (i.e., above the 90% generalization level) in the face of substantial levels of input variability, thus isolating the core challenge of object invariance without introducing confounding sources of performance-degrading factors such as background clutter. When models fail to recognize realistic images containing clutter (performance typically plateaus around 60–70%), one can never quite be sure whether the model is simply not very good, or whether it actually might be a very good model when given the benefit of figure-ground filtering that we think the biological system enjoys. Given the performance-based validation of our model on the core object recognition problem, we can then incrementally “ratchet up” the difficulty of the problem to explore how manipulations along different dimensions, like the occlusion (described in this paper) or background clutter (the subject of ongoing research to be described in a subsequent paper) affect performance.

We rendered objects with ±20° in-depth (3D) rotations (including a random 180° left-right flip for objects that are asymmetric along this dimension), and overhead lighting positioned uniformly randomly along an 80° overhead arc, to generate considerable lighting variability. Rendered images were then presented to our model with in-plane (2D) transformations of 30% translation, 20% size scaling, and 14° in-plane rotations. We assessed baseline performance of our model by reserving two exemplars per category for testing, and using the rest for training (results reflect averages over 10 random train/test splits). To capture an observer’s ability to make multiple fixations on an object, which can be used in an aggregate manner during the recognition process (Ratcliff, 1978; Bradski and Grossberg, 1995; Ratcliff and McKoon, 2008), we also examined the performance benefits that result from aggregating (majority voting) outputs over transformations of the images (see Methods for details).

The mean recognition rate on novel test items for the LVis model was 92.2% with the highest level of majority voting, which is well above the chance level of 1% for 100-way simultaneous discrimination, and indicates that the network is capable of performing quite well at the basic task of recognizing a large number of object categories in the face of extensive variability in the input images. With no voting, the generalization performance was 79.6%, and with 2D-only voting it was 86.5%.

We also developed two other comparison networks that have the same architecture as the LVis model, but lack recurrent processing mechanisms, which are used to assess the comparative impact of recurrent processing. These models used standard purely feedforward backpropagation learning (Rumelhart et al., 1986) – the error-driven learning in the Leabra model is a mathematical approximation of that in backpropagation (O’Reilly, 1996), so this is the most reasonable point of comparison for a purely feedforward network. The first backpropagation network (*Bp Distrib*) used standard parameters (i.e., 0 mean weights with 0.5 uniform random variability, learning rate of 0.01), which provided an unbiased starting point for learning and ended up producing highly distributed representations across the hidden layers, as is typical for backpropagation networks. Its performance on the object recognition test was slightly worse than the LVis model, obtaining 88.6% correct with full majority voting, 82.4% with 2D-only voting, and 77% with no voting. The second backpropagation network (*Bp Sparse*) attempted to capture the ability of the LVis model to develop relatively sparse representations due to the presence of recurrent inhibitory competition within its layers (O’Reilly, 1998). We hypothesized that strong negative initial bias weights (−3.0) and inputs that were pre-processed with the same kWTA inhibitory competition as used in the LVis inputs, would produce sparse patterns of activity across all layers and drive learning in a more robust manner. This sparse parameterization improved the performance of the backpropagation network significantly, resulting in 94.6% correct with full majority voting, 90.7% with 2D-only voting, and 86.53% with no voting. Overall, this level of performance was comparable to other standard feedforward object recognition models on this dataset, as will be reported in another publication.

### Recurrent processing under occlusion

**Figure 3:**
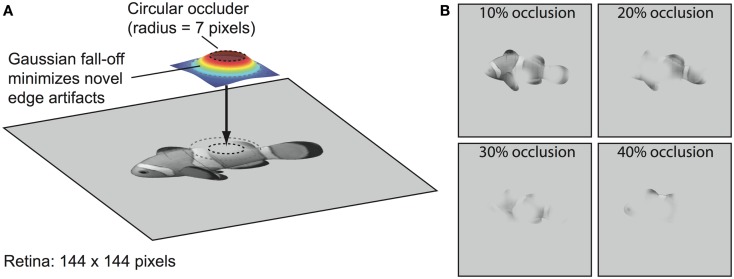
**Blob-based occlusion**. **(A)** Images were occluded by applying a filter that was set to 1.0 within a circle of radius 5% of the image size (i.e., 5% of 144 pixels or 7 pixels) and then fell off outside the circle as a Gaussian function. The final effective size of the filter was 42 × 42 pixels. The filter was used as a two-dimensional weighting function between the object and the background gray level such that image regions that fell within the circle region at the top of the filter were completely occluded with the background gray level. **(B)** Examples of different occlusion levels. Percent occlusion parameterized an equation that specified the number of times to apply the filter (see Methods). Additional occlusion examples are shown in S4.

Our first test of the role of recurrent processing in object recognition focuses on the case of partial occlusion of images. To algorithmically and parametrically manipulate occlusion in an automated fashion, we use a method similar to the “Bubbles” approach (Gosselin and Schyns, 2001) in which selected portions of an image are spatially masked via filtering operations. Specifically, we partially occluded portions of object images with varying numbers of randomly positioned circular “blob” filters softened with a Gaussian blur around the edges (Figure 3). This minimizes the introduction of novel edge artifacts, which is important given that the model does not have figure-ground mechanisms that code the ownership of each edge as belonging to the target object or the occluder (e.g., Zhaoping, 2005; Craft et al., 2007). Thus, this manipulation tests the ability to complete an underspecified input signal – which the brain undoubtedly does during occluded object recognition (Kourtzi and Kanwisher, 2001; Lerner et al., 2002; Rauschenberger et al., 2006; Weigelt et al., 2007; Wyatte et al., 2012a) – but without interference from features belonging to the occluder. This assumes there is at least partial separability of the border ownership coding and grouping- or completion-related processing, which has been suggested to be the case in the figure-ground segregation literature (Poort et al., 2012; Scholte et al., 2008). While V1- and V2-level mechanisms such as those related to illusory contour perception (Lee and Nguyen, 2001; Seghier and Vuilleumier, 2006; see also Biederman and Cooper, 1991) could potentially assist with filling-in parts of the occluded objects, with higher-levels of occlusion, there is enough visual information missing that lower-level continuation-based mechanisms would likely fail to add much. A comprehensive model of the early levels of visual processing in V1 and V2 that includes border ownership coding and illusory contour continuation would be necessary to determine the relative contribution of each of these mechanisms with realistic visual occlusion, but we argue that our methods provide a reasonable approximation for the impact of naturally occurring forms of occlusion on the upper levels of the visual pathway (e.g., V4 and IT), which are the focus of the present research.

**Figure 4:**
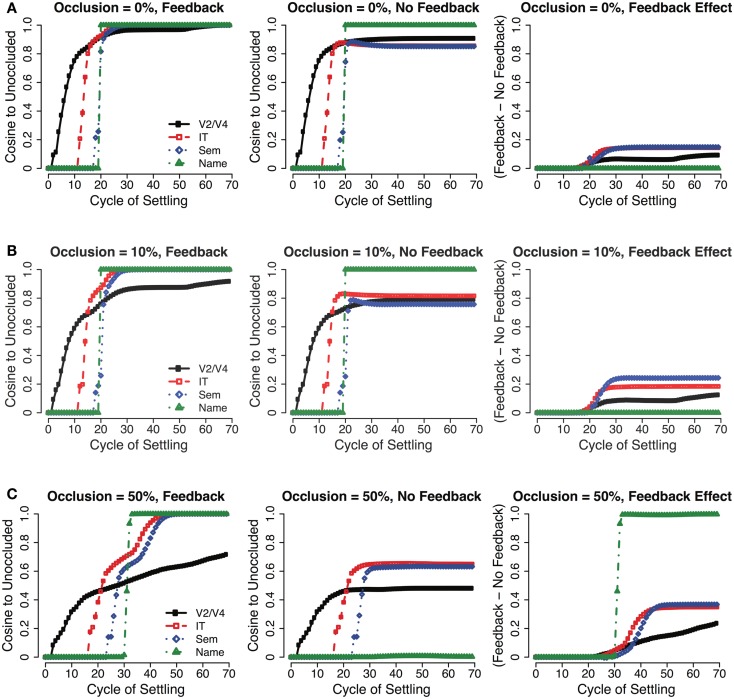
**Recurrent interactions between adjacent layers during cycles of updating for 0, 10, and 50% occlusion cases of an object**. By computing the cosine of the activity pattern for each layer compared to what would be expected when processing an unoccluded object, the network interactions that give rise to the named output can be observed. **(A,B)** When inputs are relatively unambiguous, the network converges rapidly with only a short latency between the first IT responses and activation of the correct output (ca. 10 cycles). **(C)** The correct output can still be resolved when inputs are highly ambiguous, but only after considerable recurrent interactions between layers that serve to fill in missing information reinforce the overall network state. In this case, the latency between the first IT responses and activation of the correct output is longer (ca. 15 cycles), in accordance with the recurrent interactions between layers, which take time to stabilize. Also note that the V2/V4 state does not fully complete, but the IT and Semantics patterns are identical to the unoccluded case, indicating that the higher-levels of the network complete, while the lower-levels do not (“amodal completion”). Recurrent excitatory feedback plays a critical role in this completion effect, as is shown in comparison with a network having no top-down feedback weights – this effect is more apparent with higher-levels of occlusion.

To directly measure the impact of recurrent processing in the LVis model for these partially occluded images, we assessed the extent to which the network was able to reconstruct a more complete representation of the occluded image (Figure 4). For each cycle of network activation updating during the processing of a given input image, we computed the cosine (normalized inner product) of the activity in each layer of the network compared to the final activity state of each such layer for that object when the object was unoccluded. Thus, this analysis reveals the extent to which the network is able to reconstruct over cycles of processing an internal representation that effectively fills-in the occluded parts of the image, based on prior learning about the object. To determine the role of recurrent processing in this process of reconstruction, we compared the standard LVis model with one where the strength of the top-down connections was reset to zero, thus removing the ability of higher-level representations to feed back and provide top-down knowledge of object properties based on prior learning. However, this comparison model still benefits from inhibitory recurrent processing, which we will see later plays a critical role in enhancing robustness to occlusion.

As Figure 4 shows, the recurrent connections play an important role in filling-in missing visual information, with their effect being greatest in magnitude when images are highly occluded (e.g., 50% occlusion). The IT layer in our model almost universally produces a complete object representation, with smaller completion effects observable in extrastriate layers. This finding is in accordance with object completion effects described in the literature, which indicate that their effects are largest in higher-level visual areas (e.g., IT, LOC), thus representing the *perceived* object, with lower-level areas representing mainly visual information that is present in the stimulus itself (Rauschenberger et al., 2006; Weigelt et al., 2007).

**Figure 5:**
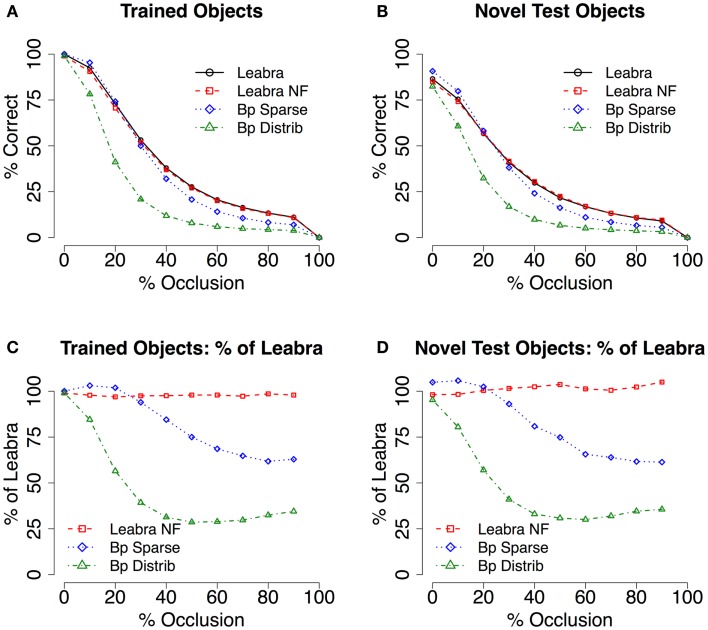
**Test of recognition under partial occlusion conditions**. **(A)** Mean recognition performance (with 2D voting – see methods and supplemental material for raw results) for trained objects, comparing full recurrent processing in Leabra with and without feedback (Leabra NF = no feedback) and purely feedforward backpropagation (Bp Sparse = sparse parameters, Bp Distrib = distributed parameters). Recurrent processing in Leabra facilitates robust recognition under partial occlusion. The Leabra model without feedback performs equivalently, suggesting that it is specifically inhibitory processing that explains this robustness. (**B)** Mean recognition for novel test objects, comparing between the same models as A. The advantage of Leabra’s recurrent connectivity is similarly apparent during generalization. (**C,D)** Results as a percentage of the Leabra performance – the slope of the lines in A and B masks the substantial effect sizes present – For trained objects, Bp Sparse performs as low as 66% compared to Leabra, and Bp Distrib as low as 31%. Again, results were qualitatively similar for novel test objects.

Next, we address the question of whether this recurrent filling-in process can actually lead to better recognition performance for occluded objects. In Figure 5, we see some indication of an advantage from the LVis networks over the backpropagation networks, especially in the case of the Bp Distrib network, which suffers dramatically from the effects of occlusion. The Bp Sparse network holds up much better, and an advantage for the LVis model is only observed for the higher-levels of occlusion, where it does become quite substantial on a percentage basis.

Given the differences in level of top-down filling-in for the intact LVis model relative to the one without top-down feedback connections, we initially expected to also see this difference reflected in the overall level of performance of these two networks. However, no such difference is evident in the results, which we have validated in multiple ways. To explain this puzzling result, it is important to ask whether in general a top-down signal can be more accurate than the bottom-up signal that activates it in the first place. Specifically, in the absence of other sources of information (e.g., from other modalities or prior context), the higher-levels of the network depend upon an initial feedforward wave of activation for their initial pattern of activity, and it is this activity pattern that then is sent back down to the lower-levels to support further filling-in. But if the initial feedforward activation is incorrect, this would presumably result in an incorrect top-down signal that would support the wrong bottom-up interpretation of the image, and thus reinforce this incorrect interpretation further. In other words, top-down support can be a double-edged sword that cuts both ways, and by recognizing this, we can understand why it does not produce a net increase in overall recognition accuracy.

To explain why the LVis model without top-down feedback connections also performs better than the Bp Sparse network at these higher occlusion levels, we attribute the advantages to the inhibitory competition present in the LVis networks that extends beyond the initial responses within a given layer. This form of recurrent inhibition dynamically adjusts to the level of excitation coming into a given layer, and thus in the highly occluded cases the inhibitory level can decrease correspondingly, enabling more activity overall to propagate through the network. In contrast, the strong negative bias weights that give rise to the sparse activities in the Bp Sparse network are in effect prior to the first responses, and thus may result in under-activation of the units for high levels of occlusion. Thus, we find evidence for the importance of recurrent inhibitory competition in providing dynamic renormalization of network response over a wide range of input signal strengths (Carandini and Heeger, 2012).

Taken together, these results show that both of the major forms of recurrence present in the LVis model can have important functional benefits: the top-down excitatory connectivity from higher areas supports filling-in of missing information compared to a network without this top-down recurrence. This could be important for many different cognitive tasks, where the missing information would be useful. However, absent other more informative sources of input, this top-down recurrence does not result in an overall improvement in recognition accuracy. Nevertheless, here we do see the advantage of the inhibitory recurrent dynamics, for renormalizing activations in the face of weaker occluded inputs.

### Recurrent connectivity and learned object representations

Another prediction from the recurrent connectivity of our model is that top-down signals should shape lower-level representations. For example, Kriegeskorte et al. (2008) showed that visual representations in inferotemporal (IT) cortex reflect semantic influences, for example, a distinction between living and non-living items. Importantly, this organizational property of IT cortex was unable to be explained in terms of bottom-up visual similarities, and was further unaccounted for by various feedforward models including those that learn “IT-level” visual features (Kiani et al., 2007). Other areas in the ventral pathway have also been shown to reflect action-based representations, possibly due to interactions with dorsal areas associated with object manipulation and tool use (Culham and Valyear, 2006; Mahon et al., 2007; Almeida et al., 2010; Mahon and Caramazza, 2011). Other evidence for top-down influences from prefrontal cortex to IT have been found during delayed responding categorization tasks (Freedman et al., 2003).

We hypothesized that these non-classical organizational properties of IT cortex are due to constraints imposed by recurrent connectivity with other neural systems over the course of learning. Simply put, recurrent connectivity allows error-driven learning signals about object properties to be circulated between neural systems, causing the similarity structure of non-visual systems to be reflected in visual areas. Semantic relationships between object categories have been suggested to be maintained by the anterior temporal pole (Patterson et al., 2007), which sends descending feedback to high-level ventral areas, and is thus a candidate structure responsible for the semantic organization observed in IT responses.

**Figure 6:**
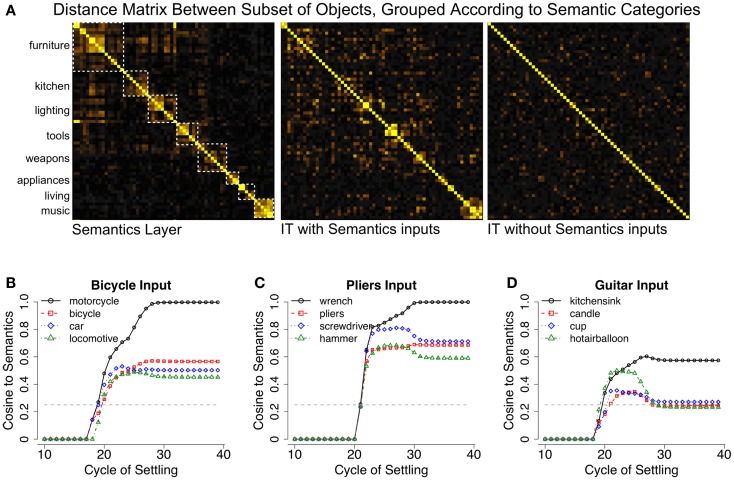
**Semantic effects in LVis**. **(A)** Top-down semantic influences on inferotemporal (IT) cortex representations in the model, in terms of distance matrix plots showing the normalized dot product (cosine) distance between semantic or IT representations (yellow = more similar). The semantics contain a categorical structure (intuitive categories indicated by dotted white squares) with some hierarchical organization, for example, among furniture, kitchen, lighting, and tools. The IT layer with semantic influences reflects a blend of these semantics and bottom-up visual similarities. The correlation between the IT layer with semantics and the actual semantics is 0.72, IT layer without semantics and the semantics is 0.57, and between the IT layers with and without semantics is 0.79. (**B)** Trajectory of the Semantics layer when a bicycle image was presented to a network that was not trained on bicycles, showing cosine similarities of the current semantics activation pattern to the canonical semantics for indicated categories. The network interprets the bicycle as a motorcycle (closest trained category), but the semantics layer representation actually has bicycle as its second closest pattern, indicating that it can infer veridical semantic properties from visual appearance. The dotted gray line indicates the mean similarity of the input semantics to the semantics of all other categories, which was 0.25 for the categories tested here. (**C)** Similar results for a pliers image, which was also not trained. (**D)** Guitars did not exhibit obvious visual similarity to semantically related trained items, and thus, the model was unable to infer their semantic properties.

We were able to demonstrate these ideas in our model by providing top-down semantic inputs to the IT layer (Figure 6A), with a similarity structure derived from pairwise similarities for each of the 100 object categories obtained from latent semantic analysis (LSA; Landauer and Dumais, 1997). Figure 6A shows that the IT layer of our model comes to reflect this semantic structure, as a result of influences from the top-down projections from semantic representations to IT. Importantly, learned object representations remain relatively distinct, and object recognition performance is unaffected. Thus, recurrent processing allows the visual properties of objects and non-visual semantic properties to be concurrently represented in the same neural substrate by simultaneously satisfying multiple bottom-up and top-down constraints during learning.

In addition to enabling our model to capture this important data, the shaping of IT representations according to semantic structure enables the model to bidirectionally map between purely visual and purely semantic similarity spaces (Gotts et al., 2011). Importantly, semantic similarity spaces have been shown to be distinctively non-visual (Kriegeskorte et al., 2008) and might very well contradict them. Thus, the relative position of IT cortex in the ventral visual hierarchy uniquely allows it to represent a balance of visual and non-visual properties and serve as an important translation point between these knowledge domains. This bidirectional perceptual-conceptual translation might underlie findings from the categorization literature in which semantic and/or conceptual knowledge about visual categories can cause them to be perceived as perceptually similar or different, regardless of their intrinsic visual similarity (Lupyan and Spivey, 2008; Lupyan et al., 2010; Lupyan, 2012). We tested our model’s ability to perform perceptual to conceptual mapping by reserving a set of 6 object categories during training (bicycle, pliers, chair, guitar, machine gun, and chandelier) and recording the semantic activation associated with these untrained categories.

Figures 6B–D demonstrates the model’s ability to produce semantic patterns that reflect the visual properties of objects from the reserved categories in relation to the other trained categories. For example, bicycles activated the semantics for motorcycle, and pliers tended to activate the semantics for either wrench or screwdriver. The resulting activation patterns were also similar to the ground-truth semantics for the untrained categories, indicating that the model could infer the veridical semantic features from visual appearance alone. Similar results were obtained for the other categories except for guitars, which failed to reliably activate semantically related items (instead, they weakly activated kitchen sink, hot air balloon, etc.). This overall pattern of results indicates that the model can infer the semantics of a novel object from its appearance, assuming the object contains visual features that are consistent with semantically related categories. Guitars presumably failed this test of semantic generalization because their visual features do not appear in other music-related categories (e.g., drums, pianos, synthesizers). Despite this failure, this finding seems reasonable – if a novel object is really quite different in appearance from known objects, like a “Greeble” (Gauthier and Tarr, 1997), it might be difficult to infer its purpose from visual properties alone.

## Discussion

We have described a biological model of the ventral visual pathway that demonstrates several important ways in which a recurrent processing architecture could contribute visual object recognition. We showed that top-down connections can fill in missing information in partially occluded images. In addition, recurrent inhibitory competition in our model contributed additional robustness in the face of high levels of occlusion, through dynamic renormalization of activation levels. We also showed how top-down connectivity shapes the learned representations in our model to reflect semantic, as well as visual, information, in agreement with recent data (Kriegeskorte et al., 2008). This dual mapping between semantic and visual information enables the network to understand the semantic implications of visual features, properly generalizing semantic information based on bottom-up visual features of novel object categories. All of these results derive from principles developed as a general theory of the neocortex (O’Reilly, 1998; O’Reilly and Munakata, 2000; O’Reilly et al., 2012), which emphasizes the importance of the brain’s ability to solve hard problems with powerful error-driven learning, and more generally specifies how relatively simple recurrent processing dynamics can give rise to more advanced cognitive phenomena.

Our results demonstrate how the dynamics that arise from recurrent connectivity can be important for vision across multiple timescales. First, these dynamics contribute in a meaningful way to the brain’s robustness to visual degradations like partial occlusion by reinforcing probable “hypotheses” about the underlying stimulus through rapid recurrent processing. For example, an image of an occluded fish will weakly activate neural populations that are tuned to *fish* features (e.g., the dorsal fin, the tail, etc.) as well as neural populations that are tuned to other visually similar, but irrelevant, features (Wyatte et al., 2012b). Our model suggests that the brain could resolve this ambiguity via excitatory top-down connections by amplifying and filling-in neurons that are tuned to additional features that are consistent with the bottom-up inputs, but may not have been present in the actual stimulus. Competitive influences are equally important, which serve to suppress spurious activations that do not constitute valid category representations. This idea has been previously described in well-understood biological models of feedforward object processing such as HMAX (Riesenhuber and Poggio, 2002; Serre et al., 2007a) which contains a maximum operation that selects the most active feature across competitors for subsequent processing. While the efficacy of the maximum operation has been explored in the context of object clutter (Riesenhuber and Poggio, 1999; see also Wyatte et al., 2012b for a similar investigation using the LVis model), it has yet to be seen whether the same operation would be useful for the partial occlusion manipulation that we have explored here in which feature activation is vastly restricted. Thus, a comparison of different types of models on occluded object recognition tasks would be useful to determine the relative importance of mechanisms such as the maximum operation, compared to top-down amplification and filling-in.

Our results indicate that the result of recurrent processing over time is a consistent, and often object-complete representation at the IT-level. We found that these recurrent dynamics could also be a double-edged sword, and did not necessarily result in overall increases in recognition accuracy despite their ability to fill in missing or ambiguous information – if the top-down signal was inaccurate, then the system could equally be led astray in its overall interpretation. Overall, these recurrent dynamics are similar to other attractor networks that “clean up” noisy representations from perceptual processing modules and produce top-down biasing effects (e.g., McClelland and Rumelhart, 1981; Mozer and Behrmann, 1990; Kveraga et al., 2007). Our results show how these same principles can be realized in a unified, large-scale model of biological object recognition operating on real visual inputs.

Recurrent inhibitory dynamics are equally important for resolving degraded inputs during object recognition. Our results suggest that the inhibitory mechanisms present in our model dynamically adjust to the amount of excitation coming into a given area, which can cause weak signals to be perceived as amplified via normalization that increases their dynamic range. Normalization has been proposed as a canonical neural computation found within many brain regions spanning multiple sensory modalities (Carandini and Heeger, 2012) and is also an integral part of recent high performance computer vision models that are loosely based on the biology of the visual system (Pinto et al., 2009, 2011). However, a neural mechanism has not been definitively associated with normalization. While our model demonstrates that this computation can be accomplished by recurrent inhibitory dynamics, other models have found similar results can be produced with excitatory feedback (Heeger, 1992, 1993). Regardless of the implementation, these results collectively point to the importance of recurrent processing mechanisms that extend past the first responses in brain areas in resolving degraded inputs during object recognition.

While the iterative recurrent processing exhibited by our model can ultimately converge on the complete pattern of neural activity that corresponds to the correct category of an occluded stimulus, this processing can take quite some time to converge when the stimulus is heavily occluded (Figure 4C, compared to Figures 4A,B). Thus, our model makes the experimental prediction that interrupting the processing of heavily occluded inputs should impair recognition more than interrupting the processing of relatively unoccluded inputs due to there being a higher probability of preventing network convergence on a stable representation. Recent psychophysical studies from our lab that use backward masking to disrupt ongoing recurrent processing are consistent with this prediction (Wyatte et al., 2012a).

Recurrent processing at longer timescales that extend across the course of learning allow disparate brain areas that project into the ventral pathway, such as higher-level semantic areas (Kriegeskorte et al., 2008), to shape perceptual representations. “IT-level” features extracted via feedforward unsupervised learning mechanisms have failed to account for these semantic influences (Kiani et al., 2007), suggesting that they represent dimensions that are not reflected in raw visual similarities. Our recurrent model accounts for this data and we also demonstrate how this higher-level organization of visual responses can be used to translate between perceptual and conceptual representations in which categories are formed according to non-visual metrics (Gotts et al., 2011).

Indeed, recent research has suggested that conceptual knowledge of visual categories can cause them to be perceived as perceptually similar or different, regardless of their intrinsic visual similarity (Lupyan and Spivey, 2008; Lupyan et al., 2010; Lupyan, 2012). What is less known, however, is whether this conceptual influence is present in perceptual representations themselves or due to a similarity metric computed by post-perceptual, decision processes (Chen and Proctor, 2012). While most data on object categorization suggest that IT cortex is tuned to shape-based properties shared across categories while neurons in prefrontal cortex represent more abstract, categorical properties (Freedman et al., 2001, 2003), recent data indicate that IT neurons do indeed exhibit abstract, categorical properties during certain timeframes of their full response (Meyers et al., 2008; Liu et al., 2009). Are these categorical properties simply feedback “echoes” from prefrontal categorization circuits or can conceptual knowledge modify the shape-based tunings of IT neurons?

Our results indicate that recurrent processing indeed modifies perceptual representations by allowing non-visual information from nearby associated brain areas to be incorporated into learning signals. This simple mechanism is likely responsible for a broad range of effects, such as action-related response properties in the ventral stream (due to connectivity with dorsal areas involved in object manipulation and tool use; Culham and Valyear, 2006; Mahon et al., 2007; Almeida et al., 2010; Mahon and Caramazza, 2011) and task-relevant IT neural tunings (due to connectivity with higher-level cognitive systems; Sigala and Logothetis, 2002; Nestor et al., 2008). Valence and emotion have also been shown affect perceptual processing, likely due to feedback from the amygdala and other limbic structures (Vuilleumier, 2005; Lim and Pessoa, 2008; Padmala and Pessoa, 2008), but so far no studies to our knowledge have investigated organizational changes in sensory areas. Overall, we suggest studies that investigate organizational structure (e.g., Kriegeskorte et al., 2008) are a fruitful domain for future research on object learning.

The detailed time course of feedforward, feedback, and inhibitory events that lead up to visual perception has been the subject of considerable debate in the literature. Research has suggested that object identity can be read out from IT neural populations in as little 80–100 ms (Oram and Perrett, 1992; Keysers et al., 2001; Hung et al., 2005) with the general conclusion that these responses must be driven solely by the initial feedforward spikes since the spikes must pass through 4 cortical areas (V1, V2, V4, and IT) with inter-areal latencies on the order of 10 ms (Nowak and Bullier, 1997). Our model is largely consistent with these feedforward latencies. For unambiguous inputs, object identity is reliably reflected in the IT activation pattern within 20 cycles (Figures 4A,B). Assuming 40–60 ms for the first spikes to appear in V1, this means 20 cycles corresponds to 40–60 ms in cortex, or around 2–3 ms per cycle. Each cycle updates the membrane potential (*V_m_*, see S2 for equations) of all model units as a function of their input conductances, and thus a latency of 20 cycles for IT readout is a reasonable extension of the biology, especially in the context of large populations of neurons where the rate code approximates the instantaneous average population firing across discrete spiking neurons (Guyonneau et al., 2004).

In addition to the well-known feedforward latencies of ventral stream areas, research has indicated that downstream areas such as prefrontal cortices categorize inputs on the order or 150 ms (Thorpe et al., 1996; Vanrullen, 2007). However, some recent estimates place the latency of recurrent processing effects well within the 100–150 ms window (Lamme and Roelfsema, 2000; Foxe and Simpson, 2002; Kveraga et al., 2007; Roland, 2010), and thus it becomes increasingly unclear whether these latencies are purely driven by feedforward responses from IT neurons or reflect substantial influence from recurrent processing mechanisms. Our model may provide some clarification of these issues. Specifically, feedback projections send information back to earlier areas as soon as it is sent forward, gradually incorporating more and more recurrent loops, and inhibitory competition influences are always present, providing online renormalization effects. Thus, we do not believe there is such a thing as *purely* feedforward processing. Instead, it is just a matter of the extent to which recurrence plays a critical role in processing. For unambiguous inputs, our model converges quickly and identity can be resolved rapidly without much influence from recurrent processing. The predominant task used in studies citing support for purely feedforward processing involves a binary decision about whether an image contains an animal (Thorpe et al., 1996; Li et al., 2002; VanRullen and Koch, 2003). Thus, our model might suggest that this “animal vs. no animal” task involves relatively little ambiguity and thus, does not critically depend on recurrent processing for success. Alternatively, this task might rapidly recruit recurrent processing in as little as 100 ms (Koivisto et al., 2011).

With highly ambiguous inputs, recurrent processing becomes increasingly important for robust object recognition. In our model, this translates to overall longer latencies for convergence (Figure 4C). Accordingly, neurophysiological recordings have suggested that ambiguity is associated with longer latencies of processing, allowing for more iterations of feedforward, feedback, and local inhibitory interactions before convergence (Akrami et al., 2009; Daelli and Treves, 2010). Whether this convergence dynamic reflects rapid dynamics within and between hierarchically adjacent areas or comparatively longer latency influence from more distant sites that reflect “top-down” processing like attention is an open question that will need to be addressed to fully understand the dynamics involved in object recognition.

Much remains to be explored in the domain of recurrent processing in visual object recognition. As noted earlier, the issue of figure-ground processing and a potential role for top-down and bottom-up interactions in this domain is a topic of current research with the LVis model, and successful resolution of these issues would help to resolve several limitations of the current model, both in terms of being able to process images with realistic backgrounds at high levels of performance, and being able to use more naturalistic forms of occlusion. More generally, there are many different ideas in the literature about how the overall object recognition process may unfold across the different visual areas, and about the potential role for recurrent processing in the brain. Thus, different models may suggest very different conclusions about the role of recurrent processing in object recognition. We are excited to compare our predictions against those of other such models, to eventually converge on a better understanding of how the biological system functions.

## Materials and Methods

### Structure of the LVis model

The LVis (Leabra Vision) model starts by preprocessing bitmap images via two stages of mathematical filtering that capture the qualitative processing thought to occur in the mammalian visual pathways from retina to LGN (lateral geniculate nucleus of the thalamus) to primary visual cortex (V1). The output of this filtering provides the input to the Leabra network, which then learns over a sequence of layers to categorize the inputs according to object categories. Although we have shown that the early stages of visual processing (through V1) can be learned via the self-organizing learning mechanisms in Leabra (O’Reilly and Munakata, 2000; O’Reilly et al., 2012), it was more computationally efficient to implement these steps directly in optimized C++ code. This optimized implementation retained the k-winners-take-all (kWTA) inhibitory competition dynamics from Leabra, which we have found to be important for successful recognition performance. Thus, the implementation can be functionally viewed as a single Leabra network.

For a full description of the early visual processing and parameters used in the model, see S1. The Leabra algorithm used to train and test the model is described in full detail in S2.

### CU3D-100 dataset

The CU3D-100 dataset consisted of 3D models from 100 diverse visual categories with an average of 9.42 exemplars per category. The individual models were downloaded from the Google 3D Warehouse^2^. Each model was normalized for differences in position, scale, and rotation using a set scripts written in Ruby and then imported into a software renderer where it was subjected to ±20° in-depth (3D) rotations (including a random 180° left-right flip for objects that are asymmetric along this dimension) with an overhead lighting positioned uniformly randomly along an 80° overhead arc. Models were rendered to PNG images in RGB color at a resolution of 320 × 320 pixels. This rendering process was repeated 20 times with different random 3D depth and lighting variations for each individual model, producing a total of 18840 images. The resulting dataset can be downloaded at http://cu3d.colorado.edu. A full breakdown of categories and number of models is available in S3.

### Training and testing methods

The model was trained for 1000 epochs of 500 images per epoch. Two exemplars per category were reserved for testing. For each image presentation, the original image was converted to grayscale and downscaled to 144 × 144 pixels and a randomly parameterized affine transformation that translated, scaled, and rotated the image was then applied. These transformations were performed via a single function, which also used neighborhood smoothing to preserve image quality as much as possible. The parameters on these transformations for any given image presentation were drawn from a uniform distribution over the following normalized parameter ranges: *scale*: 0.9–1.1 (where 1.0 means presenting the image to the model at the original downscaled resolution), *translation*: −0.15–0.15 in horizontal and vertical dimensions (where 1.0 would be moving center of image to the very top or right of the model’s inputs), *rotation*: −0.02–0.02 (where 1.0 = 2π or 360°).

Given these variations in the image presentations, no two inputs were likely to be identical over the course of training. Learning was asymptotic over the first few 100 epochs, but small improvements in generalization were observed by training for the full 1000 epochs. No evidence of overfitting was observed as a function of training duration. A total of 5 batches (training from different random initial weights and ordering of stimuli, with different train/test splits) were run using this method.

A testing trial consisted of seven presentations of a single image, with a different 2D affine transformation applied each time. For 2D voting results, a majority voting procedure integrated across these presentations to determine the final output. For higher-level voting, a second-order majority vote was then taken over the 20 testing trials with different 3D variations of each individual exemplar. All comparison models were tested using these same voting methods.

### Blob-based occlusion

The blob-based occlusion algorithm involved the construction of a filter that was set to 1.0 within a circle of radius 5% of the image size (i.e., 5% of 144 pixels or 7 pixels) and then fell off outside the circle as a Gaussian function The σ parameter of the Gaussian was also set to 5% of the image size and the final effective size of the filter was 42 × 42 pixels (Figure 3). This filter was then used as a two-dimensional weighting function to determine how much of the image should be occluded with the gray background color, with 1.0 minus this value drawn from the original image. The peak of the filter contained weights of 1.0, and thus, image areas within the peak were completely occluded with the background color, and outside of that, the image exhibited a smooth gradient out to the original image. This smooth gradient (produced by the Gaussian) was important for not introducing novel input features at the edge of the circle occluder.

The percent occlusion parameter (*O*) specified the number of times to apply the filter to an image:

(1) $$N_{apply}=\text{2}.5\text{O}\left(I_{size}∕H_{width}+1\right)+0.5$$

where *O* was in the range [0, 1], *I_size_* referred to the size of the input image in pixels, and *H_width_* referred to the width of the filter.

For testing trials that used the occlusion manipulation, a majority vote was taken across the seven 2D affine transformations of a single image only, with the occlusion mask applied prior to any transformations, to ensure that an object’s occluded features did not change across different transformations. Performance without this majority voting procedure produced the same qualitative pattern of results as seen in Figure 5 and is available in S4.

### Semantics inputs

The semantic input vectors were composed of 100 different binary unit activation patterns of which 25% were active. These patterns started out as random binary patterns, which were systematically shaped over many iterations to capture the pairwise semantic similarity between the 100 object categories as captured from the standard latent semantic analysis (LSA; Landauer and Dumais, 1997) corpus (General Reading up to 1st year College), obtained from http://lsa.colorado.edu. Generating these semantics vectors was necessary because the original LSA vectors did not contain the sparse, binary patterns required to match the kWTA inhibitory dynamics of the Leabra algorithm.

The shaping procedure was accomplished via brute-force evolution described here. For each pair of patterns, bits to flip on in common between the two patterns (thus increasing their similarity) were chosen according to a softmax function weighted by the sum of the semantic distance times other pattern’s bits. Bits were flipped in on/off pairs to ensure that kWTA constraint was preserved. Bits to flip off were chosen according to the opposite of the distance (1 minus the cosine distance). Critically, after a round of bit flipping, only those changes that increased the fit of the bit pattern distance matrix with that of the source LSA distance matrix were kept (i.e., a form of “ratcheting”).

The final mean cosine difference between the two distance matrices was 0.000597733, indicating that the patterns of similarity between the random binary bit vectors did a good job of capturing the LSA similarities.

### Comparison networks

Removing feedback from the Leabra model was achieved by simply multiplying all excitatory activation through feedback projections by zero such that the resulting input to a given layer at any point in time was limited to incoming feedforward activation.

The backpropagation networks had exactly the same layer structure and connectivity as the Leabra model, except of course for the lack of recurrent feedback connections. Both networks used cross-entropy error:

(2) $$CE=\underset{k}{\sum }t_{k}\log o_{k}+\left(1-t_{k}\right)\log\left(1-o_{k}\right)$$

(where *k* is an index over output units, *t* is the target training value, and *o* is the network output value), with an additional error tolerance of 0.05 (differences in activation below this level did not drive learning), and no momentum or weight decay. The sparse network had bias weights initialized to −3.0, which greatly reduced overall levels of initial activity. A high learning rate of 0.2 was also usable with this configuration, and this higher learning rate produced better generalization. The distributed network had bias weights initialized to 0, producing high levels of activity in the layers, and a lower learning rate of 0.01 was required to obtain converging learning. Furthermore, the distributed model did *not* use the kWTA dynamics in the V1 filter front-end processing system, to more completely capture the behavior of a system that has no sparseness-inducing inhibitory dynamics or negative biases.

Both the Leabra model without feedback and the backpropagation networks used the same majority voting procedure as the Leabra model.

## Conflict of Interest Statement

The authors declare that the research was conducted in the absence of any commercial or financial relationships that could be construed as a potential conflict of interest.

## Supplementary Material

The Supplementary Material for this article can be found online at http://www.frontiersin.org/Perception_Science/10.3389/fpsyg.2013.00124/abstract

## Footnotes

^1^http://cu3d.colorado.edu

^2^http://sketchup.google.com/3dwarehouse
